# Controlling control—A primer in open-source experimental control systems

**DOI:** 10.1371/journal.pbio.3000858

**Published:** 2020-09-10

**Authors:** Christopher James Forman

**Affiliations:** Department of Chemistry, Northwestern University, Evanston, Illinois, United States of America

## Abstract

Biological systems are composed of countless interlocking feedback loops. Reactor control systems—such as Chi-Bio (https://chi.bio/), recently published in *PLOS Biology*—enable biologists to drive multiple processes within living biological samples, using a single experimental framework. Consequently, the dynamic relationships between many biological variables can be explored simultaneously in situ. Similar multivariable experimental reactors are employed beyond biology in the study of active matter and non-equilibrium chemical reactions, in which physical systems are maintained far from equilibrium through the continuous introduction of energy or matter. Inexpensive state-of-the-art components enable open-source implementation of such multiparameter architectures, which represent a move away from expensive systems optimised for single measurements, towards affordable and reconfigurable multi-measurement systems. The transfer of well-understood engineering knowledge into the hands of biological and chemical specialists via open-source channels allows rapid cycles of experimental development and heralds a change in experimental capability that is driving increased theoretical and practical understanding of out-of-equilibrium systems across a wide range of scientific fields.

## Abstract 2

Closed loop experimental configurations can drive chemical systems to become more lifelike by pushing them out of equilibrium; similar experimental arrangements can be applied to biological research. This Primer uses the recently published Chi-Bio system to explores the emerging trend in building open source libraries of hardware which has enabled some excellent science as well as bringing the price tag down.

## Multiparameter measurement and feedback systems

Soyuz and Space X capsules allow mission specialists to reach orbit. Along similar lines, well-conceived open-source control systems and high-quality state-of-the-art components give specialists the ability to construct sophisticated experimental configurations with little to no engineering expertise, bringing domain-specific knowledge closer to the laboratory equipment design process. The demand for such capabilities has resulted in the development of core control systems into which a range of high-quality state of the art sensors, actuators, and control algorithms can be inserted with minimal effort. Instead of many separate machines optimised for single measurements—such as ultraviolet to visible (UV-Vis) light spectrometers, or microscopes—these generalised frameworks allow for simultaneous measurement and control of multiple experimental parameters, under the complete direction of the end-user. Such systems are made affordable by the diverse range of extremely cheap—yet high-quality—components available on the consumer market, from supply companies such as Mouser, RS, or Digikey.

Multiparameter experimental arrangements introduce the possibility of sophisticated feedback loops to investigate coupling between several measurable quantities. By modifying an input perturbation in response to observed system behaviour, it is possible to drive the system out of equilibrium to specific steady states and actively keep it there—like balancing a pencil on its tip. The amount of energy or matter needed to maintain such a state can be tracked, allowing characterisation of the interaction between perturbed quantities and other co-monitored quantities. Although each measurement may not be as accurate as a measurement on a dedicated system, such loss of fidelity is more than compensated for by enhanced understanding of the interactions between parameters. For example, with the Chi-Bio bioreactor, Steel and colleagues [1] made use of the sensor histidine kinase—CcaS—and its cognate response receptor—CcaR—to form an optogenetic system coupled to green fluorescent protein (GFP) expression. They were able to drive the quantity of GFP in a culture of cells so it tracked an externally defined profile. The impact of the expression of GFP on other gene circuits—coupled via cellular burden—could then be monitored at distinct wavelengths with more than sufficient accuracy and precision to understand the behaviour.

Experimental feedback loops are becoming increasingly popular in other fields such as physics and chemistry.

A beautiful example of a feedback loop experiment [2]—with relevance to biologists—contributed evidence to support the topical and beguiling idea of dissipative adaptation [3,4] from the field of non-equilibrium physics, in which time-varying energy input into a system can be used to select assembly processes. Control over the assembly of a wide range of objects—including living cells—was established far from equilibrium by regulating the flow of energy supplied by a laser. Such observations are clearly of direct relevance in the behaviour of biological cells which exist in external flows of matter or energy.

**Fig 1:**
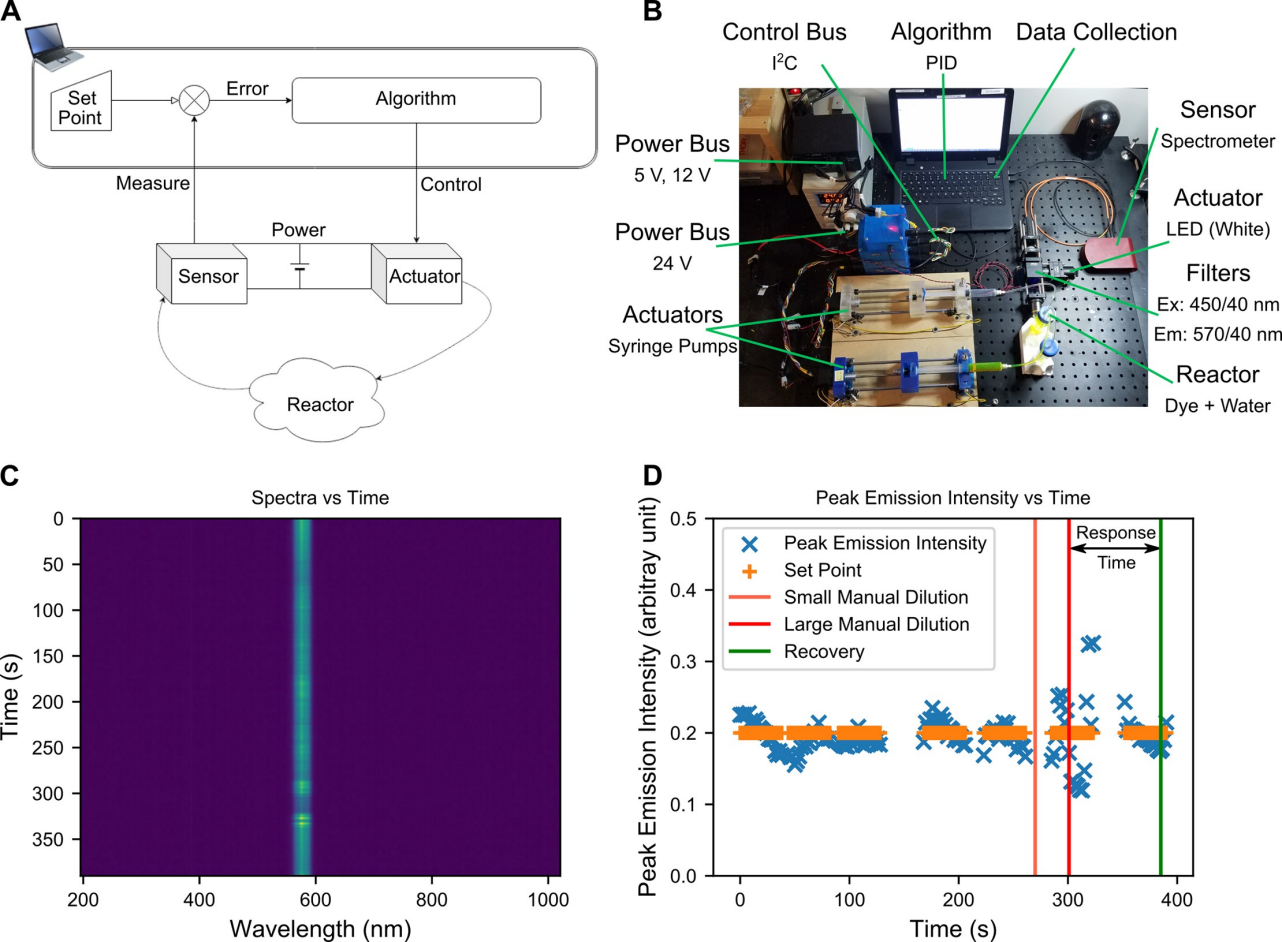
A pedagogic feedback loop for controlling dye concentration. (A) Engineered feedback loops are extremely common and well understood for driving and monitoring physical processes. A sensor and actuator work in tandem to drive an arbitrary physical process—typically using an error-driven algorithm. (B) An example feedback loop uses spectral intensity as an input into a PID algorithm to regulate the concentration of a dye (fluorescein) in a continuous flow of water. (C) Only a narrow band of the spectrum is monitored in this setup. In principle many such bands can be monitored, allowing tracking of multiple species. (D) The emission intensity oscillates about the set-point, allowing the system to respond to impulses such as the rapid manual addition of water directly into the solution. Data acquisition was intermittent to simulate lost data. The time taken to return to the steady state behaviour after a transient impulse is known as the response time. Em, Emission; Ex, Excitation; I^2^C, inter-integrated circuit; LED, light emitting diode; PID, Proportional-Integral-Derivative.

In an intriguing chemical example, a feedback loop enabled external selection of the morphology of gold nanoparticles, directing their evolution [5]. A pedagogical example is shown in Fig 1, using the author’s own setup in which a dye (fluorescein) is combined with a continuous flow of water and the resulting emission is tracked to ensure the concentration stays the same.

The concept of a feedback loop is universal (Fig 1A) and could be applied at any time and length scale. The properties of the physical system dictate the specification of the necessary hardware that drives the cost of the system. Biological and chemical processes are generally, but not always, quite slow and respond on the order of seconds to hours. For a modern processor, such timescales are easy to cope with, so simple computers such as Arduinos (Arduino, Somerville, MA), Raspberry Pis (Raspberry Pi Foundation, Cambridge, UK), or Beaglebones (Beagleboard.org Foundation, Oakland, MI) have the capacity to drive biological and chemical systems with reasonably sophisticated algorithms.

Exploring picosecond chemical process—such as photoexcitation—needs femtosecond laser pulses and substantially more expensive electronics. Consequently, re-fitting engineered bioreactors, such as Chi-Bio, for use as chemical reactors is probably more likely to work for larger macromolecular polymerisations and self-assembly reactions, which can take hours or days. Such an observation is unsurprising when you consider a cell to be, in part, a macromolecular polymerization reaction network.

To go about designing a specific reactor such as Chi-Bio, or the author’s system (Fig 1B), a range of concepts need to be understood, which are explained in Box 1. Perhaps the most enticing aspect of these kinds of system is the requirement to include software as a key part of the experiment, which often incorporates a model—such as the digital twinning process described in Box 1. Indeed, such is the scientific goal of building feedback systems: If you can understand how a physical system responds to arbitrary input, and drive it to any particular state from any other state, and you know what all its states are—both numerically and in real life—is there anything left that is learnable about that system, besides pushing it into new conditions?

**Box 1. Universal control principles: A brief definition of key concepts associated with control systems**

**Open or closed**

Control systems operate in open or closed configuration. In closed loops, the perturbing value is adjusted in response to observed behaviour, enabling stabilisation of physical quantities—such as the temperature. Open loops passively record behaviour in response to predetermined or uncontrolled input, e.g., observing turbidity as a cell culture matures or reaction colour as reagents are introduced at unregulated rates.

**Response time**

Every physical system consists of a set of processes that operate at different rates. Such a system will take time to settle to a new state in response to an input perturbation. Whether open or closed loop, the time taken for the system to respond depends on which processes are perturbed.

**Sampling rate**

The rate at which information about the physical system is captured determines the fastest process that the control system can respond to. The sampling rate should be high enough to capture information about the fastest process of interest.

**Bandwidth**

The bandwidth of the control system refers to the range of rates of processes that a control system can monitor and respond to. The wider the bandwidth, the higher the sampling rate and the faster a process that the control system can monitor and control.

**Sensitivity**

The more sensitive the control system, the smaller the detectable change in the physical system. Sensitivity is generally governed by the quality of the sensor and the amount of noise.

**Noise**

Signals that are dominated by noise can be detected by summing multiple measurements. The random noise cancels out, but the signal does not. However, control decisions must be made within a finite time, so long integration times are not always possible. Continuous accumulation of historical data is therefore extremely helpful but creates memory and processing requirements. As each new measurement arrives, it is weighed against previously collected data. Does a change in a measured quantity correspond to noise? Or is it genuinely a true change in the measured quantity of the system?

**Processing gain**

Additional signal processing can make up for poor signal-to-noise ratio. Some examples follow.

**Proportional-integral-derivative controller**

The proportional-integral-derivative (PID) controller used in Fig 1 is one example of how to maintain a steady state in a closed-loop configuration using a relatively simple algorithm. The value to apply to the actuator is calculated from the error between the desired value (the set-point) and the current sensor value. “P,” “I,” and “D” refer to 3 terms of the equation used in the calculation, which are values proportional to the error (P), the integrated sum of the error (I), and the derivative of the error (D). Often only P and I are necessary.

**Advanced algorithms**

In complex systems like spacecraft or automated factories, many sensors and actuators are monitored and adjusted continuously, so covariant effects must be considered in the model used to make control decisions. A process called digital twinning compares experimental and theoretical versions of a system to check performance. The better characterised a system becomes, the more likely a model can correctly predict system behaviour, yielding excellent knowledge of how to control the system.

## Comparison of systems

The Chi-Bio [1] is a great example of an open-source multiparameter control system, in which a team of engineers and specialists has taken care of a host of power, control, and mechanical engineering problems, enabling biologists (and chemists) to put together a wide range of experiments involving combinations of a broad variety of components. A comparison of Chi-Bio with the author’s own home-built feedback control system for polymer chemistry, dubbed Automated Parametric Explorer (APEX), reveals some key similarities and differences that will help provide a baseline of examples at different price points for anyone thinking about entering the space of multiparameter control systems.

Chi-Bio consists of a series of predesigned circuit boards that assemble to form a mechanical infrastructure that creates a cavity to host a monitored reaction chamber in standard reaction vessels. The control and power system service a range of sensors (spectrometer, thermometer) and actuators (light emitting diodes [LEDs], laser, fluidic pumps, stirrer) enabling interactions to occur within the reaction chamber volume. Up to 8 bioreactors can be operated in parallel from the same controlling computer (a Beaglebone).

APEX is part off-the-shelf and part homemade. It is mainly a software framework for linking together a wide variety of devices that are controlled via universal serial bus (USB) and a network of Arduinos. Each Arduino controls a specific laboratory actuator (such as an LED, a motor, pneumatic solenoids) which can be distributed around an opto-fluidic setup and hooked into a single control and power framework. The assembly in Fig 1 took less than a day to set up and begin acquiring data. It is trivial to upload complex functions to each Arduino allowing low-level commands to trigger complex actuation behaviour. Sensing is performed via standard devices—such as cameras and spectrometers—which employ conventional optics. A simple potentiostat has also been integrated. Such a setup transfers laboratory control into user-written software allowing all these devices to be incorporated into a single data-driven application.

### Power supply

Chi-Bio uses a standard 12 V power supply that is down-regulated to 6 V and 3.3 V. Such direct current (DC) to DC conversion is achieved with buck convertors, which are inexpensive circuits. In APEX, power supply is a standard 450 W computer power supply delivering 5 V and 12 V power rails. APEX also employs a 24 V rail that enables control over higher-power components, such as pump motors, through H-Bridge circuits. The Chi-Bio also employs a watch circuit that monitors for short circuits caused by splashes and automatically cuts power in that eventuality.

### Optical system

The Chi-Bio optical input consists of a 7-LED board that generates narrow-band light signals across the visible spectrum as well as a UV LED and a 650 nm diode laser for optical density measurements. The output is monitored by an 11-channel spectrometer chip that consists of a single light-sensitive array with a checker-board pattern of 11 filters. Thus, each region of the sensor chip monitors a slightly different frequency, trading resolution for number of channels. Chi-Bio monitors laser brightness through the sample and—since the LED optical path is perpendicular to the spectrometer’s optical axis—Chi-Bio can monitor fluorescence emissions caused by one of the 8 excitation LEDs without the need for expensive dichroic mirrors and filters.

In contrast, APEX employs individual LEDs mounted on threaded 1-inch discs (SM1CP2M, Thorlabs, Newton, NJ) enabling their simplified incorporation into a standard 1-inch optical setup. A high-power broadband LED can be filtered using narrow-band filters to achieve a wide range of input signals, which can be focused using normal optics as shown in Fig 1B. Two bandpass filters and a dichroic are used to isolate emission and excitation using the same objective lens. This design helps to keep optics and fluids well separated. The signals across a range of wavelengths and integration times can be detected by a spectrometer (CCS250 from Thorlabs, Newton, NJ). To put the full-blown optical solution into contrast with Chi-Bio, the precision milled 1-inch disc alone costs around $20, a basic optical filter set starts at $1,000, and the spectrometer $2,000+. While the optical arrangements and quality of signals are no doubt superior for APEX, the cost difference is enormous. The spectrometer in Chi-Bio is a single chip that costs $9 but still provides good enough results to make excellent scientific progress. The value is in the flexibility and number of simultaneous channels. The entire Chi-Bio kit costs around $800 and measures temperature, absorbance, and fluorescence. APEX can be a fluorimeter, a microscope, a spectrometer, a potentiostat, a camera, a fluidic control system, or a machine-learning and data management centre, and it probably comes in at around $20,000 for parts—but not design labour—which is about the price of a high-end UV-Vis instrument.

### Control protocols

In both cases, the same universal protocol enables communications across the devices in the system. Several low-level standards exist such as inter-integrated circuit (I^2^C) and serial peripheral interfaces (SPIs), in which one wire provides a clock to synchronize data transfer and the other wires provide one or more data channels and secondary circuit activation selection mechanisms. The I^2^C system used in Chi-Bio and APEX is an industry standard that is used to link together microchips with just 2 wires. Typically, these are primary-secondary configurations in which a single primary can control many secondary circuits.

## Conclusion

As open-source systems employ ever more sophisticated architectures, the gaps between the knowledge of biologists, engineers, physicists, and chemists dwindle to occupy a similar realm, in which inert matter is pushed out of equilibrium to bring it one step closer to living material.

## Abbreviations

APEX: Automated Parametric Explorer
DC: direct current
GFP: green fluorescent protein
I^2^C: inter-integrated circuit
LED: light emitting diode
PID: proportional-integral-derivative
SPI: serial peripheral interface
USB: universal serial bus
UV-Vis: ultraviolet to visible
